# Supplementary figures and images for: Knockdown of the Sodium/Potassium ATPase Subunit Beta 2 Reduces Egg Production in the Dengue Vector, Aedes aegypti
Source: Insects. 2023 Jan 5;14(1):50. doi: 10.3390/insects14010050 (PMC9862990; doi:10.3390/insects14010050)

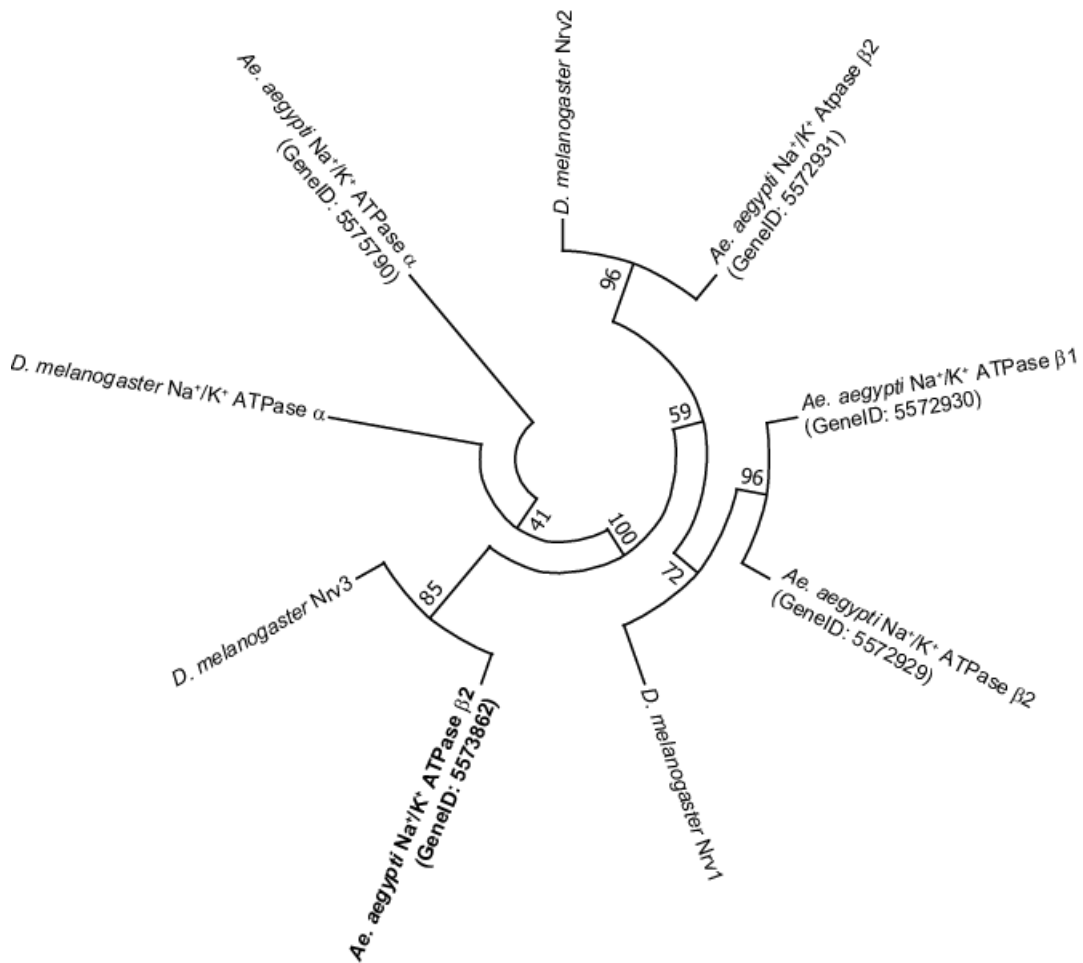

Supplement: Supplementary file 1 [file insects-14-00050-s001.zip › insects-2085502-supplementary.pdf]
